# Dietary walnut oil modulates liver steatosis in the obese Zucker rat

**DOI:** 10.1007/s00394-013-0573-z

**Published:** 2013-08-13

**Authors:** Anja Fink, Corinna E. Rüfer, Julie Le Grandois, Alexander Roth, Dalal Aoude-Werner, Eric Marchioni, Achim Bub, Stephan W. Barth

**Affiliations:** 1 Department of Physiology and Biochemistry of Nutrition, Max Rubner-Institut, Haid-und-Neu-Str. 9, 76131 Karlsruhe, Germany; 2 Department of Safety and Quality of Fruit and Vegetables, Max Rubner-Institut, Haid-und-Neu-Str. 9, 76131 Karlsruhe, Germany; 3 Aerial, Parc d’Innovation, rue Laurent Fries, B.P. 40443, 67412 Illkirch-Cedex, France; 4 Equipe de Chimie Analytique des Molécules BioActives, Faculté de Pharmacie, IPHC, Université de Strasbourg, 74 route du Rhin, 67400 Illkirch, France

**Keywords:** NAFLD, Lard, SFA, MUFA, PUFA

## Abstract

**Purpose:**

Non-alcoholic fatty liver disease (NAFLD) is the hepatic manifestation of the metabolic syndrome. We aimed to clarify the impact of dietary walnut oil versus animal fat on hepatic steatosis, representing the initial step of multistage pathogenesis of NAFLD, in Zucker obese rats.

**Methods:**

Zucker lean ad libitum (a.l.), Zucker obese a.l. or Zucker obese pair fed (p.f.) to the lean received isocaloric diets containing 8 % walnut oil (W8), W14 or 14 % lard (L14) (*n* = 10/group). Body weight, clinical serology, liver weight, lipid content and fatty acid composition and hepatic lipid metabolism-related transcripts were evaluated.

**Results:**

Compared to lean, Zucker obese a.l. and p.f. showed hepatic triacylglyceride (TAG) accumulation. In Zucker obese p.f., W14 compared to W8 and L14 reduced liver lipids, TAG as well as hepatic omega-6 (n-6)/n-3 ratio and SCD activity index [(C18:0 + C18:1)/C18:0 ratio] paralleled by decreased lipoprotein lipase mRNA in obese p.f. and elevated microsomal triglyceride transfer protein mRNA in lean and obese. Further, W14 elevated the fasting blood TAG and reduced cholesterol levels in obese.

**Conclusions:**

In our model, consumption of W14 inhibited hepatic lipid accumulation along with modulated hepatic gene expression implicated in hepatic fatty acid influx or lipoprotein assembly. These results provide first indication that dietary lipids from walnut oil are modulators of hepatic steatosis as the initial step of progressive NAFLD pathogenesis.

## Introduction

Non-alcoholic fatty liver disease (NAFLD) is a common public health problem worldwide with an increased prevalence during the last three decades [1–3]. The prevalence of this disorder is strongly associated with several risk factors of the metabolic syndrome (MetS) such as obesity, dyslipidemia, hypertension and type 2 diabetes [4–7]. Therefore, NAFLD is regarded as the hepatic manifestation of the MetS [1, 8]. NAFLD, a multistage and progressive disease, ranges from lipid accumulation in hepatocytes (hepatic steatosis) to hepatic steatosis with a necroinflammatory component (non-alcoholic steatohepatitis; NASH) and might finally progresses to liver cirrhosis [5, 6, 9].

Liver triacylglyceride (TAG) levels are the result of the balance between influx of fatty acids, *de novo* lipogenesis, TAG synthesis and delivery by very low density lipoprotein (VLDL) assembly [10–13]. Thus, the accumulation of TAG in the steatotic liver reflects a difference between the rate at which fatty acids reach hepatocytes, or are synthesized therein, and the rate at which they are metabolized, stored or assembled [10, 12, 13]. Further, this initial expansion of ectopic lipid levels during steatosis development tends to be associated with progressive metabolic dysregulation and, in particular, altered hepatic insulin resistance and the development of inflammation.

Until now, there has no specific therapy been developed to directly treat the pathogenesis and progression of NAFLD. Instead, indirect approaches are pursued by treating major risk factors of NAFLD. However, this treatment strategy is complex, because not a single but various risk factors influence and associate the induction and progression of NAFLD. Among others, mainly the developing insulin resistance and lipid dysmetabolism promote the pathological hepatic TAG accumulation [5, 6]. Hence, the cornerstones of the therapy as well as prevention of NAFLD are to ameliorate the metabolic risk factors by reducing body weight and improving insulin sensitivity and hyperlipidemia [6, 9].

The enhanced prevalence of NAFLD is also driven by changes of dietary habits and primarily increased consumption of dietary fat. Recent studies have further indicated that not solely the quantity of dietary fat, but also fatty acid composition is a risk-determining factor for NAFLD: Patients with NAFLD not only showed a higher intake of saturated fatty acids (SFA), but also increased omega-6 (n-6) polyunsaturated fatty acids (PUFA) consumption while n-3 PUFA uptake is reduced, leading to a significant shift toward increased dietary n-6/n-3 ratio [3, 8, 14].

Recent literature provides indications that these dietary changes might also lead to endogenous alterations in the hepatic fatty acid composition toward an increase in the n-6/n-3 PUFA ratio [15, 16], which associates with the development of an adverse metabolic profile and contribute to the pathogenesis of NAFLD [16]. Further, animal studies have demonstrated that an excess of n-6 PUFA in the liver is associated with a proinflammatory [3, 17] as well as steatogenic state with pronounced hepatic fat accumulation [18–20]. Given these observations, a dietary approach changing the n-6/n-3 PUFA ratio in the diet might be effective enough to change hepatic n-6/n-3 PUFA ratio in a way that positively influence NAFLD-related hepatic pathogenesis [21–23].

Nuts and seeds share a fatty acid profile which is characterized by low amounts of SFA and high content of monounsaturated fatty acids (MUFA) and PUFA [24]. Among the numerous nut varieties, recent research has focused on walnuts (*Juglans regia*). One reason for this might be the “optimal” 4:1 ratio of n-6/n-3 PUFA and additionally the high content of tocopherols (mainly γ-tocopherol), phytosterols, polyphenolic antioxidants (ellagitannins) and fiber [25, 26]. With regard to a significant bioactivity, recent investigations have shown that a walnut-rich diet improved hyperlipidemia [26–30], type 2 diabetes [31, 32], cardiovascular disease [33–35], ameliorated the antioxidative status [36, 37] and provided general parenchyma-protecting effects in the liver [30, 38]. However, to our best knowledge, no data are published regarding bioactivity of walnut on NAFLD mainly focusing on the initial step of liver steatosis.

Thus, the aim of the present study was to investigate the effect of different amounts of walnut oil versus lard on hepatic lipid metabolism in a rodent steatosis model and identify hepatic pathways which might be implicated in any observed bioactivity. Therefore, we have targeted hepatic lipid content, serum lipids and sICAM, as an artherogenic and proinflammatory marker. Additionally, we focused on the expression of genes involved in hepatic lipid metabolism to explore underlying mechanisms.

## Materials and methods

### Phospholipid analysis of walnut oil

Standard solutions of soy lecithin-mix standard (certified: 14.41 % phosphatidylcholine (PC), 12.06 % phosphatidylethanolamine (PE) and 9.64 % phosphatidylinositol (PI)) from Spectral Service GmbH (Cologne, Germany) were prepared at six concentrations between 0.1 and 1.1 mg/mL. Sand (Fontainebleau) was provided by VWR (Strasbourg, France).

The walnut oil (Öhlmühle Walz, Oberkirch, Germany) has been produced by a traditional two-step process performed in a hydraulic press driven by water power. The first step consists on a cold press of walnut seeds at 100 bar, followed by a 400 bar step at 40 °C. In subsequent to the pressing procedure, the oil is filtered through a rough filter mesh and bottled. For phospholipid (PL) analysis, 75 mg of oil samples were dissolved in 1 mL of CHCl_3_, transferred onto a preparative silica gel column (35 cm × 2 cm i.d., 15 g of Si 60 silica gel, particle size 40–63 μm, Geduran, Merck) which was pre-activated with 10 mL of CHCl_3_. Elution was performed at a flow rate of 4 mL/min. First, neutral lipids such as triacylglycerols and carotenoids were removed by 250 mL of CHCl_3_. Then, PL were eluted with 200 mL of CH_3_OH/1M aqueous formic acid (adjusted to pH = 3 with triethylamine) (98:2, v/v) mixture. The solvent was removed by rotary evaporation, and the residue containing pure PL was stored at −20 °C for the chromatographic separation.

A chromatographic system, made of a 616 controller, a 2424 ELS detector and a 717 Plus autosampler (Waters, Saint-Quentin-Fallavier, France) controlled with Empower 2 software (Waters), was used to analyze PL classes. High-purity nitrogen from a nitrogen generator (Domnik Hunter, Villefranche-sur-Saône, France) was used as a nebulizing gas at a pressure of 310 kPa. The drift tube temperature was set at 45 °C. PL were separated into their respective classes (PE, PI, PC) using a 150 × 3 mm, 3 μm Luna normal phase column (Phenomenex, Le Pecq, France). The flow rate of mobile phase was 0.5 mL/min, and separations were performed at room temperature using a 20-min linear gradient ranging from CHCl_3_/CH_3_OH (88/12, v/v) to CHCl_3_/CH_3_OH/1M aqueous formic acid (adjusted to pH = 3 with triethylamine) (28/60/12, v/v/v). Each extract was dissolved in a mixture chloroform/methanol (2/1, v/v), filtered through a 0.45-μm filter (Macherey–Nagel, Hoerdt, France) to eliminate particles and injected (20 μL) in the chromatographic system. PL classes were identified by comparing their retention times with those obtained under the same analytical conditions with standards. Quantification of each PL class was performed based on a quadratic model of external calibration obtained by using standard solutions.

### Phytosterol analysis of walnut oil

Solvents used for extraction and purification were of analytical grade. Ethanol (95 %), diethyl ether, n-hexane and cyclohexane were purchased from LPCR (Strasbourg, France). Analytical-grade potassium hydroxide (LPCR, Strasbourg France) was used for saponification. 1-methyl imidazole (Sigma-Aldrich, Saint-Quentin-Fallavier, France) and *N*-methyl-*N*-trimethylsilyl-heptafluorobutyramide (MSHFBA) (Sigma-Aldrich, Saint-Quentin-Fallavier, France) were used for sterols silylation. Sterols analysis was adapted from EN ISO 12228 [39].

One mL of internal standard (betulin solution at 1 mg/mL) is added to 250 mg of walnut oil in a round-bottom flask. This sample is dissolved in 5 mL of potassium hydroxide, 0.5 M in ethanol (95 %) and heated under reflux for 15 min. After cooling, the whole saponified sample is subjected to column chromatography on an alumina column (10 g) preconditioned with 20 mL of ethanol 95 %. Elution of unsaponifiable matter was first performed with 5 mL ethanol, then with 30 mL diethyl ether. Eluents were combined and evaporated under vacuum. Residue was dissolved in 1 mL cyclohexane/diethyl ether (9:1, v/v) before being subjected to solid-phase extraction on Chromabond SiOH cartridge (3 mL, 500 mg, Macherey–Nagel, Hoerdt, France) preconditioned with 5 mL cyclohexane. Washing step was performed with 5 mL of cyclohexane/diethyl ether (9:1, v/v). Finally, sterols elution was achieved using 8 mL of cyclohexane/diethyl ether (1:1, v/v). The eluent was evaporated under vacuum. Sterols were silylated using 50 μL of 1-methyl imidazole and 1 mL MSHFBA, before injection (1 μL) in GC-FID. Separation was made by a CP Sil 8-CB column (50 m, 0.25 mm, 0.25 μm, Agilent Technologies). Helium of high purity (99.9995 %) was used as carrier gas at a flow rate of 1.4 mL/min. The injector was set to 250 °C and the flame ionization detector to 320 °C. The column was set at 80 °C, held for 1 min, and raised to 280 °C (rate of 10 °C/min). The temperature was then raised to 308 °C (rate of 0.5 °C/min) and then to 320 °C (rate of 2 °C/min). The final temperature was maintained for 3 min. Peaks were identified by a comparison with standards and to relative retention times (RRT). Quantification was performed using betuline as internal standard, considering response factors between sterols and betuline as equal to 1.

### Tocopherol analysis of walnut oil

The method used for analysis was adapted from Skrivanova et al. [40]. About two grams of walnut oil were diluted in 25 mL of HPLC grade n-hexane (LPCR, Strasbourg, France). The solution is filtered through a 0.45 μm PTFE membrane (Macherey–Nagel, Hoerdt, France) before injection. Separation of tocopherols (α-, β-, χ- and δ-tocopherol) is performed on a Lichrospher Si 60 column (250 × 4.6 mm, 5 μm, Merck, Darmstadt, Germany) with an isocratic mobile phase based on n-hexane/2-propanol (98:2, v/v). Detection was done by fluorimetry, with excitation and emission wavelengths set to 284 and 330 nm, respectively. Quantification of each tocopherol was performed using a calibration curve prepared with tocopherol standards (Sigma-Aldrich, Saint-Quentin-Fallavier, France).

### Animals and diets

**Fig. 1:**
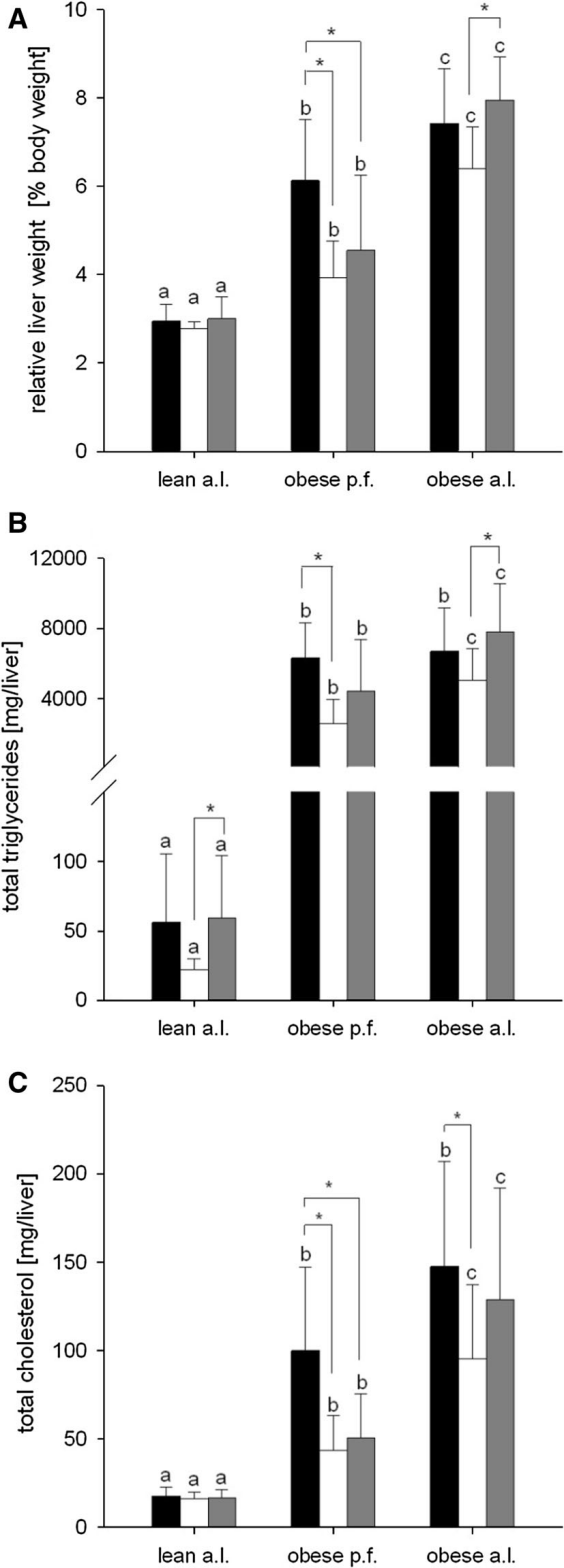
Effects of different diets with 8 % walnut oil (*black*), 14 % walnut oil (*white*) or 14 % lard (*gray*) on **a** relative liver weight, **b** hepatic TAG and **c** hepatic cholesterol content in lean ad libitum (a.l.), obese pair fed (p.f.) or obese a.l. Zucker rats. Data are expressed as mean ± SD (*n* = 9–10). The letters *a*, *b* and *c* describe the significant differences (*p* < 0.05) of the phenotypes with the same feeding. Significant differences within phenotypes are labeled by *asterisk* (*p* < 0.05)

**Fig. 2:**
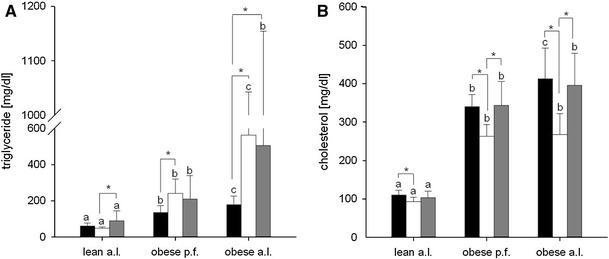
Effects of different diets with 8 % walnut oil (*black*), 14 % walnut oil (*white*) or 14 % lard (*gray*) on **a** serum TAG content and **b** serum cholesterol content in lean ad libitum (a.l.), obese pair fed (p.f.) or obese a.l. Zucker rats. Data are expressed as mean ± SD (*n* = 9–10). The letters *a*, *b* and *c* describe the significant differences (*p* < 0.05) of the phenotypes with the same feeding. Significant differences within phenotypes are labeled by *asterisk* (*p* < 0.05)

Obese (fa/fa; *n* = 60) and lean (Fa/+; *n* = 30) female Zucker rats were purchased from Charles River Laboratories (Lyon, France) at the age of 6 weeks. All animals were housed in a temperature- and humidity-controlled animal facility under ambient temperature of 21 ± 2 °C, 55–65 % of relative humidity and a 12–12 h light–dark cycle. During the first week of adaption, all rats were provided ad libitum (a.l.) tap water and a standard experimental diet AIN93G with 7 % corn oil (Ssniff, Soest, Germany). In subsequent to the adaptation, rats were randomly allocated to one of the following groups: lean a.l., obese pair fed (p.f.) or obese a.l. The group of the obese p.f. rats were fed the same amount of food consumed at the previous day by the age-matched lean counterparts. This pair feeding concept has been conducted, because the total energy intake is a major determinant for obesity (Table 5), dyslipidemia (Fig. 2a) as well as hepatic steatosis (Fig. 1a, b). As the pair feeding group receives the same amount of isocaloric diets and thus the same amount of energy compared to the lean group, this confounding factor has been controlled by this concept. Thus, any observed differences between lean and obese p.f. are determined by genotype, while differences between obese p.f. and obese a.l. are due to the different intake of amount of food. All groups were further randomly subdivided into one of the three intervention groups (*n* = 10) receiving either an isocaloric diet based on AIN93G containing 8 % (w/w) walnut oil (W8; Öhlmühle Walz, Oberkirch), 14 % walnut oil (W14) or 14 % (w/w) lard (L14) (ssniff, Soest, Germany) for 10 weeks (Table 1). The fatty acid composition of diets and the analytical data of walnut oil phytosterols, tocopherols and phospholipids are summarized in Tables 2 and 3, respectively. Food intake was measured daily and body weight four times a week. At the end of the feeding period, the animals were fasted overnight, deeply anesthetized by carbon dioxide and killed by decapitation. Liver tissues were collected and stored at −80 °C. All animal experiments were approved by the Animal Care Committee of the Regional Administrative Authority, Karlsruhe (35-9185.81/G-89/09), and all animal care and handling were conducted in accordance with the guidelines of the German law on animal care.

**Table 1:** Composition of experimental diets (%; w/w)

Experimental diets	8 % Walnut oil	14 % Walnut oil	14 % Lard
Casein	20.00	20.00	20.00
Corn starch mod	38.74	23.74	23.74
Maltodextrin	13.20	13.20	13.20
Saccharose	10.00	10.00	10.00
Lignocellulose	5.00	0.30	0.30
l-cystein	0.30	0.30	0.30
Vitamin-AIN	1.00	1.00	1.00
Mineral nutrients	2.50	3.50	3.50
Butylhydroxytoluol	0.01	0.01	0.01
Choline	0.25	0.25	0.25
Fat	8.00	14.00	14.00
ME, Atwater (MJ/kg)	16.60	16.60	16.60

**Table 2:** Fatty acid composition of experimental diets (% of total fatty acids)

Fatty acid methyl esters (% of total fatty acids)	8 % Walnut oil	14 % Walnut oil	14 % Lard
C12:0 (lauric acid)	0.0	0.0	0.2
C14:0 (myristic acid)	0.2	0.1	2.6
C16:0 (palmitic acid)	9.8	9.4	34.7
C17:0 (margaric acid)	0.1	0.1	0.7
C18:0 (stearic acid)	5.8	5.8	20.5
C20:0 (arachidic acid)	0.1	0.1	0.1
C22:0 (behenic acid)	0.0	0.0	0.0
∑ SFA	16.0	15.5	58.7
C14:1 (myristoleic acid)	0.0	0.0	0.2
C16:1 (palmitoleic acid)	0.2	0.2	2.8
C18:1n-9t (elaidic acid)	0.1	0.1	0.2
C18:1n-9c (oleic acid)	27.5	27.3	26.9
C20:1 (gondoic acid)	0.3	0.3	1.2
C24:1n-9 (nervonic acid)	0.0	0.0	0.1
∑ MUFA	28.1	27.9	31.3
C18:2n-6t (linoelaidic acid)	0.3	0.3	0.0
C18:2n-6c (linoleic acid)	39.2	39.6	8.8
C18:3n-6 (γ-linolenic acid)	0.5	0.5	0.3
C18:3n-3 (α-linolenic acid)	15.9	16.1	0.5
C20:2n-6 (eicosadienoic acid)	0.0	0.0	0.2
C20:3n-6 (dihomo γ-linolenic acid)	0.0	0.0	0.1
C20:3n-3 (eicosatrienoic acid)	0.0	0.0	0.0
C20:5n3 (eicosapentaenoic acid)	0.0	0.0	0.0
∑ PUFA	56.0	56.5	9.9
n-6/n-3 PUFA ratio	2.5:1	2.5:1	18:1

**Table 3:** Analysis of phytosterols, tocopherols and phospholipids in walnut oil (mg/100 g)

Walnut oil	(mg/100 g)
Brassicasterol	0.00
Campesterol	8.89
Cholesterol	0.39
d5-Avenasterol	1.09
d7-Avenasterol	0.00
d7-Campesterol	0.00
d7-Stigmasterol	49.46
Sitostanol	17.91
Sitosterol	163.48
Stigmasterol	n.d.
Phytosterols total	241.21
α-Tocopherol	2.394
γ-Tocopherol	45.450
δ-Tocopherol	5.044
Tocopherols total	52.890
Phosphatidylethanolamine	22.5
Phosphatidylinositol	n.d.
Phosphatidylcholine	n.d.
Phospholipids total	22.5

*n.d.* not detectable

### Blood parameters

Blood was collected into serum monovettes (Sarstedt, Nümbrecht, Germany), and serum was prepared from clotted blood by centrifugation (2,500×*g*, 10 min) and stored at −30 °C until further analysis. Serum cholesterol (CHOD-PAP, Roche) and TAG (GPO-PAP, Roche) were measured using enzymatic assay kits. Serum level of soluble intercellular adhesion molecule (sICAM)-1 (R&D systems, Wiesbaden, Germany) was measured using a rat ELISA kit.

### Liver lipid and fatty acid analyses

Lipid extraction of 2 g liver tissues was carried out according to Hara and Radin [41] with slight modifications. The liver tissue was homogenized in 18 mL of hexane/isopropanol (3/2; v/v) (Carl Roth, Karlsruhe, Germany) containing 0.01 % butylhydroxytoluol (BHT; Merck, Darmstadt, Germany). After sonification and addition of 12 mL aqueous sodium sulfate (Merck), samples were placed on a horizontal shaker for overnight extraction. The samples were centrifuged and hexane overlayer was removed to a fresh pre-weighted vial. The remainder bottom layer was again extracted with 18 mL of hexane/isopropanol (7/2; v/v) containing 0.01 % BHT, and after centrifugation, the hexane overlayer was given to the vial already containing the hexane layer of the initial extraction. After evaporation under a stream of nitrogen gas, total lipid content within sample vials was weighted for gravimetric estimation of total liver fat. Fatty acids were analyzed by gas chromatography (GC). Fatty acid methyl esters (FAME) were prepared by transesterification of total lipids with TMSH. 50 mg of the lipid extract dissolved in 1 mL dichloromethane and aliquots of 10 μL were used for FAME analysis. After addition of 100 μL of 90 mg/L methyl-nonadecanoate as internal standard, 30 μL of TMSH, as well as 70 μL methanol containing 1 % BHT, the organic extract was evaporated to complete dryness under a stream of nitrogen. The residue was dissolved in 100 μL TMSH and 500 μL methanol, stirred in the dark overnight and subjected to GC analysis. GC analysis was carried out on a gas chromatograph with mass spectrometric detection (GC-MS-QP-2010 Ultra, Shimadzu, Kyoto, Japan) using split/splitless injection. Chromatographic separation of FAME were achieved on a fused silica capillary column with a non-bonded cyanopropyl-type phase (SP-2560, 75 m × 0.18 mm ID, 0.14 μm film thickness, Supelco, Taufkirchen, Germany) using a helium carrier gas flow of 0.9 mL/min and a linear temperature gradient (100 °C for 5 min, then 4 °C/min to 230 °C and hold at 230 °C for 25 min). The injector port temperature was set to 230 °C. The temperatures of transfer line and ion source were set to 250 and 200 °C, respectively. The injection volume was 1 μL with a split of 1:5. Individual methyl esters were identified and quantified using a standard mixture of 37 FAME (37 Component FAME Mix, Supelco, Taufkirchen, Germany). As based on the fatty acid quantification, hepatic stearoyl CoA desaturase (SCD-1) activity index was calculated by the (C18:0 + C18:1)/C18:0 ratio. Hepatic cholesterol and TAG were measured as previously described for serum samples.

### Real-time quantitative PCR

Total RNA was isolated from the liver tissue using a commercial kit according to the manufacturer’s instruction (Total RNA and protein isolation kit, Macherey–Nagel, Düren, Germany). cDNA was prepared by reverse transcription of 2 μg total RNA using the Transcriptor First Strand cDNA Synthesis Kit and oligo(dT) primers (Roche). Samples were stored at −20 °C until further use. Semiquantitative real-time PCR was carried out using the Light Cycler480^®^ Instrument (Roche). Primer and probe sequences were designed by Universal Probe Library (Roche) as listed in Table 4. The reaction mixture contained 5 μL cDNA, corresponding to 50 ng total RNA, 0.5 μM of each primer, 1 μM probe and Light Cycler480^®^ Probe Master Mix (2× conc.) (Roche). The PCR conditions were as follows: 10 min of initial denaturation at 95 °C followed by 45 amplification cycles each at 95 °C for 10 s, 60 °C for 30 s and 72 °C for 1 s with a terminal cooling period of 10 s at 40 °C. The analysis was carried out with the Light Cycler480^®^ Software (Roche) using the relative quantification ∆∆CT-method and normalized by beta-actin as reference gene.

**Table 4:** Primer sequences and probes used for quantitative real-time PCR

Genes	Forward primer	Reverse primer	UPL no.	Genbank accession no.
ACTB	cccgcgagtacaaccttct	cgtcatccatggcgaact	#17	NM_031144.2
ACC1	acagagatggtggctgatgtc	gatccccatggcaatctg	#4	NM_022193.1
ChREBP	aatcccagcccctacacc	ctgggaggagccaatgtg	#10	AB074517.1
DGAT2	gctggtgccctactccaag	agcttagggacggtgatgg	#9	NM_001012345.1
FASN	ggccacctcagtcctgttat	agggtccagctagagggtaca	#6	NM_017332.1
GPAm	tccagacaccacatcaagga	ctcctccatgactcaacgtg	#127	NM_017274.1
LPL	atgatgtggccaggttcatc	gggctccaagactgtacccta	#20	L03294.1
PPAR-α	tgcggactaccagtacttaggg	gctggagagagggtgtctgt	#116	NM_013196.1
PPAR-γ	ggtgaaactctgggagatcct	aatggcatctctgtgtcaacc	#115	NM_013124.2
SREBP1	gtacagcatggctgggaac	ggctgagcgatacagttcaa	#1	AF286470.2

### Statistical analysis

Due to high heterogeneity of variances between lean and obese, a statistical model, namely, generalized least squares ANOVA (GLS-ANOVA) was chosen that is capable of handling unequal variances for the different groups. The independent variables for the two-factorial ANOVA were “phenotype” and “food”, resulting in a 3 × 3 factorial model. As a first step, we calculated a simple model which assumes equal variances within factors “phenotype” and “food”. Subsequently, this simple model was then modified by adjusting the variance structure for “phenotype” and “food”. A likelihood ratio test was used to determine if the variance-adapted models fitted better than the simple model. When main effects or interactions were found significant, a post hoc test (Tukey–Kramer) for pairwise comparisons was applied. The* p* values that were given in the text and figures result from these post hoc tests. To test for the assumptions that have to be met for the GLS-ANOVA, we made plots to visually inspect residuals. Studentized residuals were plotted against fitted values to assess homoscedasticity. QQ-plots were used to test normal distribution of the residuals. Further, both plots were used to identify outliers. All calculations were carried out by R 2.15.2 [42]. GLS-ANOVAs were calculated by R package nlme [43].

## Results

### Analysis of dietary lipids and experimental diets

Fatty acid spectra of walnut oil and lard-containing diets were characteristic for the respective lipid. While the PUFA linoleic and α-linolenic are the major fatty acids in walnut oil, lard mainly contained the SFA palmitic and stearic acids. The resulting ratio of n-6/n-3 PUFA were 2.5:1 and 18:1 in walnut oil and lard-containing experimental diets, respectively (Table 2).

The analytical results of walnut oil phospholipid content (Table 3) showed significant concentrations only for phosphatidylethanolamine (22.5 mg/100 g), while no trace of phosphatidylinositol was recovered, and phosphatidylcholine, the major phospholipid in common foods, was present only in trace amounts and therefore was not quantified here. Further, sitosterol (163 mg/100 g) and d7-stigmasterol (49.5 mg/100 g) represent the major phytosterol constituents in walnut oil, while content of γ-tocopherol (45.5 mg/100 g) was highest compared to α-(2.4 mg/100 g) and δ-tocopherols (5.0 mg/100 g) (Table 3).

### Body weight and food intake

As shown in Table 5, body weight, weight gain and food intake were significantly affected by genotype as well as the feeding regimen, namely the pair feeding versus ad libitum in obese rats. Obese p.f. and obese a.l. rats showed a significantly (*p* < 0.001) higher initial and final body weight than lean rats with obese a.l. rats weighing more than obese p.f. rats (*p* < 0.001). Further, the mean body weight gain was significantly lower in lean than in obese a.l. (*p* < 0.001) and obese p.f. (*p* < 0.001) Zucker rats and obese p.f. rats gained less weight throughout the experiment when compared to obese a.l. (*p* < 0.001). The mean food intake during the entire experiment was approximately 25 % higher in obese a.l. than in lean (*p* < 0.001) and the matched obese p.f. rats (*p* < 0.001). As a result of the established pair feeding regimen, statistical evaluation between the lean and obese p.f. showed equal food intake between obese p.f. and lean rats.

**Table 5:** Body weight, weight gain and food intake during the experiment in lean ad libitum (a.l.), obese pair fed (p.f.), and obese a.l. Zucker rats receiving diets with 8 % walnut oil (W8), 14 % walnut oil (W14) and 14 % lard (L14)

	Experimental diets	Lean a.l.	Obese p.f.	Obese a.l.
Initial body weight (g)	W8	136 ± 7^a^	189 ± 10^b^	196 ± 13^b^
	W14	139 ± 7^a^	196 ± 8^b^	203 ± 10^b^
	L14	140 ± 9^a^	191 ± 8^b^	198 ± 11^b^
Final body weight (g)	W8	246 ± 19^a^	349 ± 21^b^	395 ± 21^c^
	W14	245 ± 20^a^	356 ± 34^b^	406 ± 36^c^
	L14	242 ± 15^a^	337 ± 27^b^	386 ± 38^c^
Weight gain (g/week)	W8	11.2 ± 1.3^a^	16.7 ± 1.7^b^	20,3 ± 1.7^c^
	W14	11.0 ± 1.6^a^	16.8 ± 3.4^b^	20.3 ± 3.5^c^
	L14	10.6 ± 1.4^a^	15.3 ± 2.8^b^	18.8 ± 3.6^c^
Food intake (g/day)	W8	13.2 ± 0.4^a^	13.2 ± 0.1^a^	16.4 ± 1.2^b^
	W14	12.8 ± 0.6^a^	13.2 ± 0.1^a^	16.3 ± 0.9^b^
	L14	13.3 ± 0.3^a^	13.4 ± 0.1^a^	16.5 ± 0.5^b^

Data are expressed as mean ± SD (*n* = 9–10). Values in a common row of the identical intervention labeled by different letters are significantly different between phenotypes

Significant differences within phenotypes are labeled by * (*p* < 0.05 compared to W8)

Besides the observed dependency of general parameters on genotype and dietary energy intake, the amount and quality of different dietary fats did not significantly influence the food intake, final body weight and mean weight gain (Table 5).

### Liver lipids

Irrespective of pair or ad libitum feeding, the Zucker obese rats developed liver steatosis as reflected significantly by higher liver weights as well as liver total lipid and TAG content as compared to the lean. In detail, the relative liver weight (Fig. 1a) and TAG contents (Fig. 1b) in obese p.f. and a.l. rats were significantly higher than in lean. Pair feeding resulted in a significantly lower relative liver weight (*p* < 0.05) when compared to ad libitum fed obese rats (Fig. 1a). With the exception of the W8 group, the hepatic TAG contents in pair fed were lower than in ad libitum fed rats (Fig. 1b). Moreover, the liver tissue of obese rats contained significantly higher (*p* < 0.01) cholesterol than the liver of lean rats, and obese a.l. rats showed higher cholesterol contents than obese p.f. rats, except of the W8 group (Fig. 1c).

In lean rats, none of the different dietary fats led to significant group differences regarding the relative liver weight or any of the analyzed lipid parameters in the liver. However, in obese p.f. rats, consumption of both 14 % fat diets significantly reduced (*p* < 0.05) the relative liver weight when compared to W8. Further, the liver weight in W14 obese a.l. rats was significantly reduced (*p* < 0.001) compared to L14 rats (Fig. 1a).

In accordance to the remainder liver parameters, also, the total liver lipid content was unaffected by any diet in lean Zucker rats. However, feeding W14 compared to W8 significantly reduced (*p* < 0.01) the total liver lipid content in obese p.f. rats. This drop in liver lipids seems to be specific to walnut, as it was not observed in L14 (data not shown). Further, the observed reduction in lipid content by W14 is a consequence mainly of the reduced hepatic TAG as the liver TAG content in obese p.f. rats was significantly reduced by W14 (*p* < 0.001) but not by L14 as compared to W8 group. In lean and obese a.l. rats, a W14 diet led to a significantly (*p* < 0.05) lower hepatic TAG compared to the L14 group (Fig. 1b). The hepatic cholesterol content was also affected by the different lipid diets. In obese p.f. rats, W14 and L14 diets reduced the hepatic cholesterol content significantly (*p* < 0.01) compared to the W8 group, and obese a.l. W14 rats had also a lower cholesterol (*p* < 0.05) content compared to the W8 group (Fig. 1c).

### Liver fatty acids

The fatty acid patterns of the experimental diets were distinct and characteristic to walnut oil and lard as detailed in Table 2. Walnut oil has a low content of SFA and a high content of PUFA, especially n-3 PUFA, whereas a high content of SFA and a low content of PUFA are characteristic for lard (Table 2).

Irrespective of the different experimental diets, obese p.f. and obese a.l. Zucker rats generally showed significantly lower (*p* < 0.001) hepatic SFA- and PUFA-contents compared to the lean Zucker rats, while the MUFA content was significantly higher (*p* < 0.001) in obese p.f. and a.l. rats (Table 2). In accordance to the higher MUFA content in obese livers, also, the SCD activity index [(C18:0 + C18:1)/C18:0 ratio] was significantly higher in obese animals (Fig. 4).

Further, the different diets differentially affected the SFA, MUFA and PUFA content in the liver of the respective feeding groups. As depicted in Table 6, the hepatic SFA levels were unaffected by any diet in lean, obese p.f. and obese a.l. Although not statistically significant, the hepatic MUFA content tended to be decreased by W14 while MUFA content was highest under L14 feeding in all phenotypes. Accordingly, also the SCD activity index significantly decreased by W14 in obese p.f. and obese a.l., while L14 compared to W14 significantly increased the SCD activity index in all phenotypes (Fig. 4). While MUFA was highest in L14 groups, feeding L14 significantly decreased PUFA content compared to respective W8 and W14 groups. Further, in obese p.f. and obese a.l., but not in lean Zucker rats, the W14 diet significantly increased (*p* < 0.001) the hepatic PUFA content compared to the respective W8 and the L14 groups. In detail, the W14 diet elevated the n-3 PUFA α-linolenic acid (C18:3n-3), eicosapentaenoic acid (C20:5n-3) and docosahexaenoic acid (C20:6n-3) in the liver of obese p.f. and obese a.l. rats compared to rats with a W8 and L14 diet (not shown). In consequence, the ratio of n-6/n-3 decreased, although n-6 increased too. This drop in n-6/n-3 ratio by W14 was also observable in the lean but not as pronounced as in obese (Table 6).

**Table 6:** Fatty acid profile in liver samples from lean ad libitum (a.l.), obese pair fed (p.f.) and obese a.l. Zucker rats receiving diets with 8 % walnut oil (W8), 14 % walnut oil (W14) and 14 % lard (L14)

Fatty acid methyl ester (% of total fatty acids)	Experimental diets	Lean a.l.	Obese p.f.	Obese a.l.
SFA	W8	52.7 ± 5.6^a^	41.9 ± 1.7^b^	40.7 ± 0.9^c^
	W14	52.7 ± 5.2^a^	41.4 ± 1.3^b^	39.0 ± 0.9^c^
	L14	52.9 ± 3.1^a^	43.0 ± 3.5^b^	41.1 ± 1.7^c^
MUFA	W8	11.1 ± 5.7^a^	48.3 ± 0.6^b^	48.8 ± 1.5^b^
	W14	7.7 ± 1.6^a^	40.1 ± 3.4^b^	39.6 ± 5.2^b^
	L14	22.1 ± 6.5^a^	51.5 ± 4.8^b^	54.2 ± 3.1^b^
PUFA	W8	36.2 ± 3.2^a^	9.8 ± 2.0^b^	10.6 ± 1.1^b^
	W14	38.6 ± 3.6^a^	18.6 ± 2.9^b,^*	21.3 ± 4.5^b,^*
	L14	25.0 ± 6.7^a,^*^,#^	5.5 ± 3.4^b^*^,#^	4.7 ± 1.8^b^*^,#^
n-6 PUFA	W8	29.9 ± 1.7^a^	9.2 ± 1.8^b^	9.9 ± 0.8^b^
	W14	32.2 ± 2.4^a^	16.6 ± 2.2^b,^*	19.1 ± 3.3^b,^*
	L14	21.9 ± 5.3^a,^*^,#^	5.3 ± 3.3^b,^*^,#^	4.6 ± 1.7^b,^*^,#^
n-3 PUFA	W8	6.0 ± 1.6^a^	0.6 ± 0.2^b^	0.6 ± 0.2^b^
	W14	6.0 ± 1.4^a^	1.9 ± 0.6^b,^*	2.1 ± 1.3^b,^*
	L14	3.0 ± 1.5^a,^*^,#^	0.2 ± 0.1^b,^*^,#^	0.2 ± 0.1^b,^*^,#^
n-6/n-3 PUFA ratio	W8	5:1	15:1	17:1
	W14	5:1	9:1	9:1
	L14	7:1	27:1	23:1

Data are expressed as mean ± SD (*n* = 9–10). Values in a common row of the identical intervention labeled by different letters are significantly different between phenotypes

Significant differences within phenotypes are labeled by * (*p* < 0.05 compared to W8) or ^#^ (*p* < 0.05 compared to W14)

### Blood parameters

**Fig. 3:**
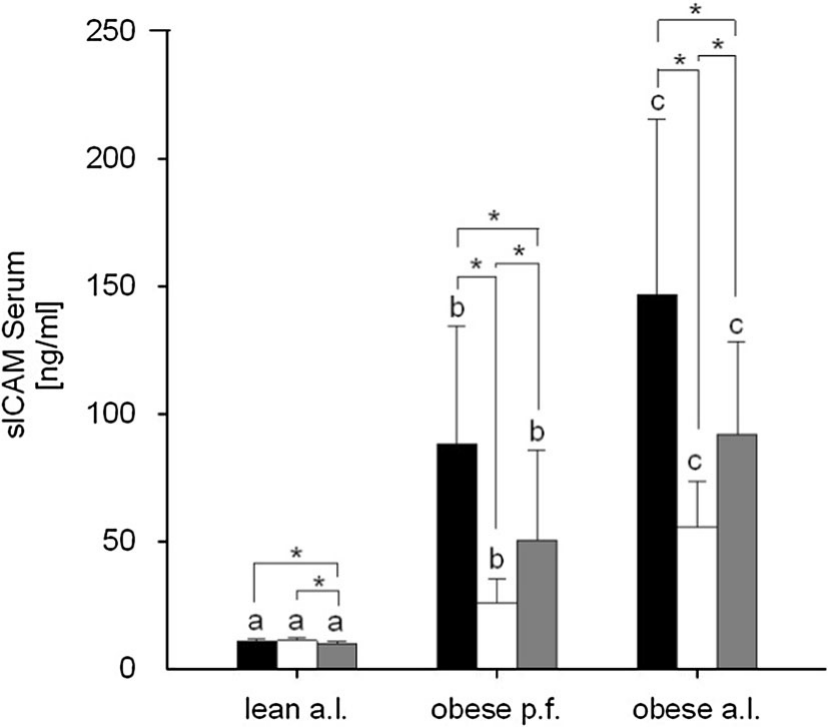
Effects of different diets with 8 % walnut oil (*black*), 14 % walnut oil (*white*) or 14 % lard (*gray*) on serum sICAM concentration in lean ad libitum (a.l.), obese pair fed (p.f.) or obese a.l. Zucker rats. Data are expressed as mean ± SD (*n* = 9–10). The letters *a*, *b* and *c* describe the significant differences (*p* < 0.05) of the phenotypes with the same feeding. Significant differences within phenotypes are labeled by *asterisk* (*p* < 0.05)

As expected, obese p.f. and obese a.l. Zucker rats developed a significant dyslipidemia reflected by a pronounced hypertriglyceridemia and hypercholesterolemia as compared to the lean (Fig. 2). Further, serum sICAM concentrations were significantly enhanced in obese p.f. and a.l. compared to lean Zucker rats, and obese a.l. showed significantly increased sICAM levels compared to obese p.f. (Fig. 3).

When compared to the W14-mediated lipid- and TAG-lowering effect in the liver of obese animals, walnut oil showed the opposite effect in plasma: In obese rats, W14 led to a significant (*p* < 0.001) increase in fasting plasma TAG. This TAG-enhancing effect seems to be not specifically deserved to the intake of W14, but also L14 showed a significant (*p* < 0.05) rise in plasma TAG when compared to W8 (Fig. 2a) in obese a.l. rats. The cholesterol-lowering effect is also apparent in lean and seems to be specific to walnut oil as solely W14, but not L14 decreased plasma cholesterol compared to W8 (Fig. 2b). Besides the modification of plasma lipid metabolites, also, circulating sICAM levels have been affected by the different experimental diets. In both obese groups, W14 and L14 led to a significant reduction in serum sICAM concentrations compared to respective W8, while W14-mediated sICAM was significantly lower when compared to L14 (Fig. 3).

Spearman’s rank correlation indicated that the total liver lipid content is positively and significantly correlated with serum sICAM (*ρ* 0.97; *p* < 0.001), while liver and serum TAG are negatively correlated (*ρ* −0.49; *p* < 0.001).

**Fig. 4:**
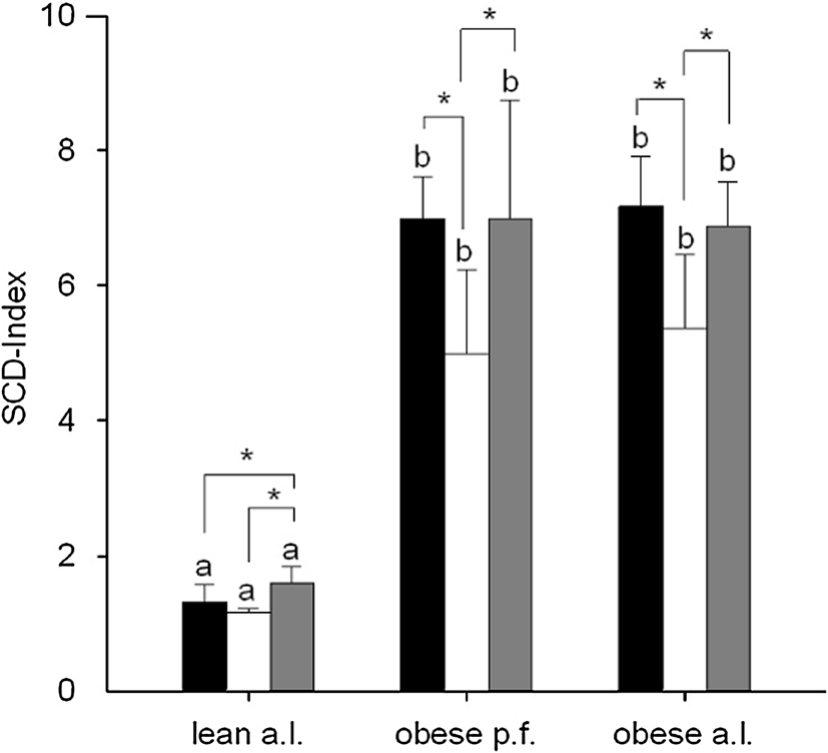
Hepatic SCD activity index [(C18:0 + C18:1)/C18:0 ratio] after feeding lean ad libitum (a.l.), obese pair fed (p.f.) or obese a.l. Zucker rats with different diets 8 % walnut oil, (*black*), 14 % walnut oil (*white*) or 14 % lard (*gray*). Data are expressed as mean ± SD (*n* = 9–10). The letters *a*, *b* and *c* describe the significant differences (*p* < 0.05) of the phenotypes receiving the same diet

### Hepatic gene expression

Transcripts coding for key enzymes and transcription factors of the lipid metabolic pathway respecting factors which influence the fatty acid uptake, lipogenic and lipolytic regulators as well as transcripts coding for factors implicated in VLDL assembly were analyzed.

**Fig. 5:**
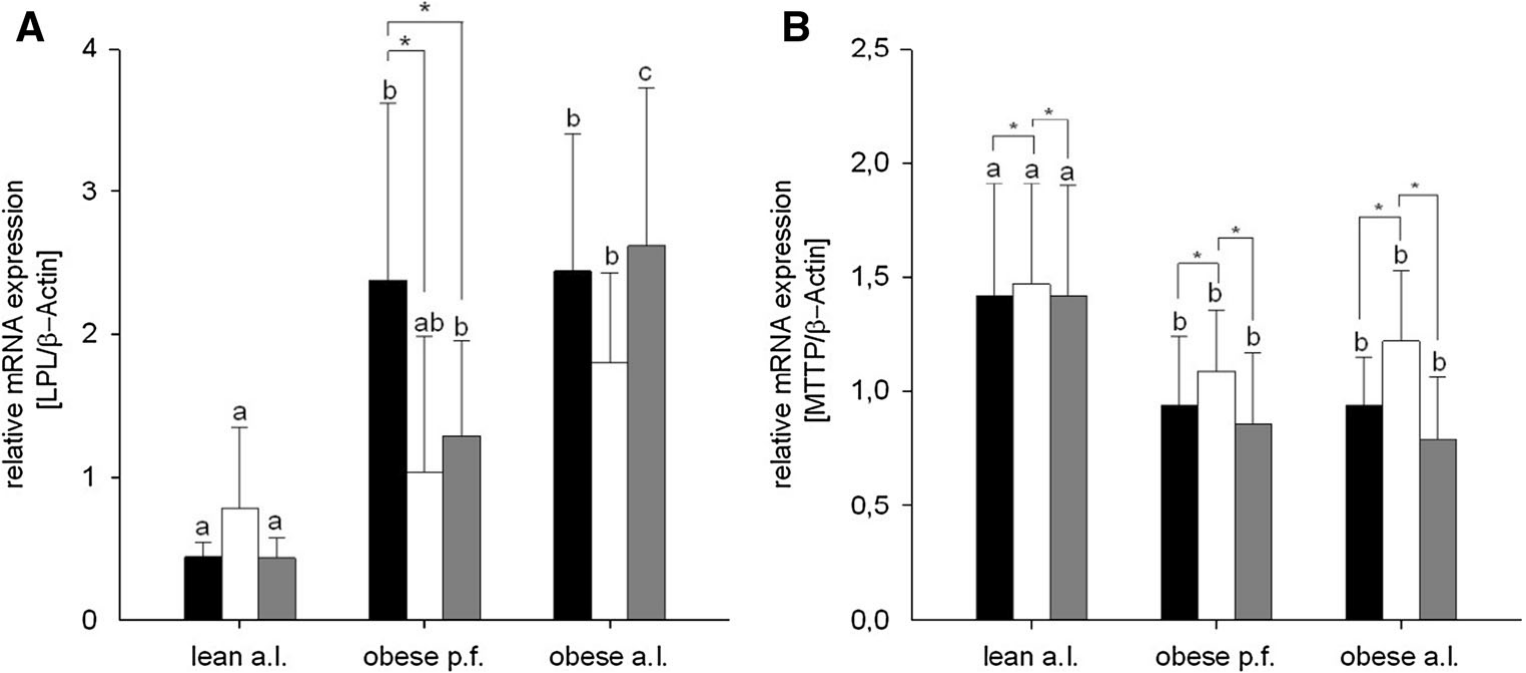
Hepatic (**a**) LPL and (**b**) MTTP gene expression after feeding lean ad libitum (a.l.), obese pair fed (p.f.) or obese a.l. Zucker rats with different diets 8 % walnut oil, (*black*), 14 % walnut oil (*white*) or 14 % lard (*gray*). Data are expressed as mean ± SD (*n* = 9–10). The letters *a*, *b* and *c* describe the significant differences (*p* < 0.05) of the phenotypes receiving the same diet

The gene expression of hepatic enzymes involved in hydrolysis of TAG to free fatty acids, namely lipoprotein lipase (LPL) showed significant phenotype differences between obese and lean Zucker rats. The liver LPL gene expression was up-regulated in obese p.f. and a.l. Zucker rats, except for obese p.f. W14, compared to respective lean groups (Fig. 5). The expression of peroxisome proliferator-activated receptor (PPAR)-α, a transcription factor involved in fatty acid oxidation was significantly higher in obese a.l. rats than in obese p.f. rats (Table 7). Amount of transcript coding for lipogenic enzymes acetyl-CoA carboxylase (ACC) and fatty acid synthase (FAS) showed significantly enhanced mRNA levels in obese p.f. Zucker rats compared to lean and obese a.l Zucker rats (Table 7). Furthermore, lipogenic transcription factors PPAR-γ and sterol regulatory element-binding protein (SREBP)-1c were up-regulated, whereas carbohydrate responsive element-binding protein (ChREBP) was down-regulated in obese Zucker rats compared to the lean (Table 7). Amount of mitochondrial glycerol-3-phosphate acyltransferase (GPAm) mRNA, which codes for a key enzyme implicated in TAG esterification, was significantly higher in obese p.f. rats compared to obese a.l and lean rats. Diglyceride acyltransferase (DGAT), a key enzyme for the esterification of TAG, was significantly induced in lean rats compared to obese a.l. rats. Furthermore, the expression of microsomal TAG transfer protein (MTTP), a key factor regulating hepatic TAG export by VLDL synthesis and assembly, was significantly down-regulated in obese p.f. and a.l. compared to lean Zucker rats. With the exception of the obese p.f. W14 group, the hepatic ICAM gene expression was significantly up-regulated in all obese compared to lean Zucker rats (Table 7).

**Table 7:** Expression of key genes involved in hepatic lipid metabolism was determined by real-time PCR in liver samples lean ad libitum (a.l.), obese pair fed (p.f.) and obese a.l. Zucker rats receiving diets with 8 % walnut oil (W8), 14 % walnut oil (W14) and 14 % lard (L14)

	Experimental diet	Lean a.l.	Obese p.f.	Obese a.l.
ACC/β-Actin	W8	2.9 ± 2.8^a^	3.3 ± 1.7^b^	2.0 ± 0.6^a^
	W14	2.7 ± 2.6^a^	5.4 ± 1.8^b^	2.8 ± 1.1^a^
	L14	3.4 ± 3.2^a^	5.2 ± 2.4^b^	2.3 ± 1.1^a^
ChREBP/β-Actin	W8	1.3 ± 0.5^a^	0.7 ± 0.2^b^	0.6 ± 0.1^c^
	W14	1.2 ± 0.3^a^	0.8 ± 0.2^b^	0.7 ± 0.1^c^
	L14	1.1 ± 0.4^a^	0.7 ± 0.2^b^	0.6 ± 0.2^c^
DGAT/β-Actin	W8	1.2 ± 0.3^a^	0.8 ± 0.3^a,b^	0.9 ± 0.4^b^
	W14	1.3 ± 0.4^a^	1.4 ± 0.5^a,b^	1.0 ± 0.4^b^
	L14	1.4 ± 0.4^a^	1.3 ± 0.6^a,b^	0.9 ± 0.5^b^
FAS/β-Actin	W8	1.7 ± 2.1^a^	2.7 ± 1.8^b^	1.6 ± 0.7^a^
	W14	1.7 ± 1.7^a^	4.9 ± 1.8^b^	2.1 ± 0.8^a^
	L14	2.0 ± 1.8^a^	4.3 ± 1.8^b^	1.9 ± 0.7^a^
GPAm/β-Actin	W8	0.9 ± 0.2^a^	2.2 ± 1.9^b^	1.3 ± 0.5^c^
	W14	1.2 ± 0.8^a^	5.0 ± 2.3^b^	1.7 ± 1.2^c^
	L14	1.2 ± 0.6^a^	3.2 ± 2.8^b^	1.2 ± 0.7^c^
ICAM/β-Actin	W8	0.6 ± 0.1^a^	1.6 ± 0.4^b^	1.6 ± 0.4^b^
	W14	0.8 ± 0.3^a^	1.2 ± 0.2^a^	1.9 ± 0.7^b^
	L14	0.6 ± 0.2^a^	1.3 ± 0.3^b^	1.6 ± 0.3^b^
PPAR-α/β-Actin	W8	2.0 ± 0.6^a,b^	1.8 ± 0.6^a^	2.3 ± 0.6^b^
	W14	2.0 ± 0.7^a,b^	1.8 ± 0.8^a^	2.6 ± 1.0^b^
	L14	2.1 ± 0.7^a,b^	1.5 ± 0.4^a^	2.1 ± 0.5^b^
PPAR-y/β-Actin	W8	1.2 ± 0.6^a^	6.4 ± 4.1^b^	5.2 ± 1.6^b^
	W14	1.1 ± 0.4^a^	5.3 ± 2.1^b^	6.5 ± 4.3^b^
	L14	1.1 ± 0.5^a^	5.8 ± 2.2^b^	5.9 ± 2.6^b^
SREBP1c/β-Actin	W8	0.8 ± 0.4^a^	1.7 ± 1.1^b^	1.9 ± 0.6^b^
	W14	1.1 ± 0.4^a^	2.3 ± 0.8^b^	2.0 ± 0.7^b^
	L14	1.1 ± 0.7^a^	2.0 ± 0.8^b^	2.0 ± 0.8^b^

Data are expressed as mean ± SD (*n* = 8–10). Values in a common row of the identical intervention containing labeled by different letters are significantly different between phenotypes

Significant differences within phenotypes are labeled by * (*p* < 0.05 compared to W8) or ^#^ (*p* < 0.05 compared to W14)

The intervention with the different experimental diets differentially modulated the expression of LPL in liver tissue. In obese p.f., both diets containing 14 % fat content led to a significant reduction of LPL mRNA in the liver compared to obese p.f. W8 Zucker rats. In contrast, no effects were observed in lean Zucker rats (Fig. 5). Spearman’s rank correlation showed that the total liver lipid content is positively and significantly correlated with the hepatic LPL mRNA content (*ρ* 0.785; *p* < 0.001).

Irrespective of the phenotype, the MTTP gene expression has been significantly increased in all W14 groups as compared to groups receiving W8 or L14 diets (Fig. 5). All other transcript levels of ACC, FAS, SREBP-1c, ChREBP, PPAR-γ, PPAR-α, GPAm, DGAT, MTTP, ICAM were unaffected by any diet (Table 7).

## Discussion

The key finding of this study stated that the quality and the quantity of dietary lipids differentially influence hepatic and circulating lipid metabolites in vivo. In detail, a high intake of walnut oil decreased hepatic TAG while fasting serum TAG levels were increased in obese Zucker rats. The decreased hepatic TAG concomitantly appeared with significant changes in fatty acid patterns in the liver and a reduced SCD activity index as well as reduced/normalized n-6/n-3 ratio in the liver. These qualitative and quantitative changes in lipid contents were associated with decreased hepatic LPL mRNA in obese p.f. rats and increased MTTP mRNA irrespective of the phenotype. Finally, a diet high in walnut oil significantly reduced the artherogenic and inflammatory marker sICAM in obese Zucker rats.

The development of efficient (diet-based) prevention and therapeutic options for NAFLD is based on a clear understanding of the etiology and mechanisms of this condition, which in turn is limited by the quality of the study model. NAFLD in lab rodents can be induced and promoted by a wide variety of factors, initially leading to changes in hepatic lipid deposition. These include diets which are high in fat or carbohydrates (e.g., fructose) or methionine and choline deficient (MCD) diets. The diet-induced NAFLD models are completed by various genetic models; the most commonly used are represented by the leptin and/or leptin receptor variants. All these currently available animal models of NAFLD are associated with various drawbacks in that they do not or only partially reflect the real picture of human NAFLD in terms of etiology, pathogenesis and disease mechanisms. For example, most of the diet-induced NAFLD models also implicate potential confounding factors such as high energy intake (by high-fat and/or high-carbohydrate diets) or the high-(fructose, lipid) or deficient-(MCD) intake of particular macronutrients. On the other side, genetic models carrying a single mutation do not share the same etiology as multifactorially/multigenetically generated human NAFLD, although clinical parameters related to NAFLD show identical aberrations [44–48]. Based on this knowledge about respective model-associated advantages and disadvantages, we chose the obese Zucker rat, integrated this model into an energy-controlled pair feeding design and further used isocaloric diets irrespective of the different fat contents. According to this experimental design, the pair feeding groups received the amount of diet adjusted to the respective lean a.l. group which received approximately 75 % of dietary calories as compared to the obese a.l. fed groups. This concept combined with isocaloric diets irrespective of fat content enabled us to analyze a “pure” intervention effect of dietary lipid quality and/or quantity on NAFLD-associated metabolic aberrations largely excluding the mentioned potential confounders such as variable energy intake.

The present study shows that an isocaloric diet with a high content of walnut oil (14 % w/w) not solely mediated anti-steatotic effects in obese rats, but it also influenced the hepatic fatty acid composition.

It has been described that various dietary fats with different fatty acid compositions differentially affect the hepatic TAG accumulation [49, 50]. In vivo studies have already shown that dietary fats from fish oil rich in long-chain n-3 PUFA resulted in a reduction in hepatic TAG concentration [49–52], which has recently been shown to be associated with a preferred incorporation of long-chain n-3 PUFA into hepatic phospholipids [53]. Further, Chechi et al. [2] have demonstrated that a flax oil diet, rich in α-linolenic acid, also reduced the hepatic TAG in obese SHR/NDmcr-cp rats. However, as recently published in ApoE-deficient mice, a diet containing 20 % (w/w) walnut oil did not change liver lipid content [54], which might be due to the complex functional impairments of this rodent model. Besides its artherogenic effect, the ApoE deficiency also results in impaired VLDL assembly [55, 56], which might be the mechanism by which walnut oil in our study mobilized and reduced the hepatic lipid content as discussed below.

While low levels of hepatic n-3 PUFA are associated with the development of liver steatosis in NAFLD patients [18, 57], a supplementation with n-3 PUFA decreased hepatic TAG content in vivo [23, 57]. Based on these data, we suggest that the observed bioactivity of high walnut oil intake in obese rats might be caused by rather than randomly associated with the observed increased levels of hepatic PUFA, especially n-3 PUFA. The predominating n-3 PUFA of walnut oil, α-linolenic acid (Table 2), is endogenously converted into eicosapentaenoic acid (EPA, C20:5n-3) and docosahexaenoic acid (DHA, C22:6n-3) by the Δ-6- and Δ-5-desaturase and elongase-5 and -2 enzyme systems, respectively. Although the efficacy of this conversion of α-linolenic acid into EPA and DHA is not quantitative, but has been estimated below 21 % [58, 59], significant two to fourfold enrichment of hepatic EPA or DHA by W14 compared to W8 was obvious (data not shown) and paralleled by a general improvement of n-6/n-3 ratio under anti-steatotic feeding with walnut oil.

Epidemiological studies have reported that besides its impact on hepatic lipid homoeostasis, a low n-6/n-3 PUFA ratio is also capable of improving the blood lipid profile [22, 51]. Riediger et al. [22] reported decreased plasma TAG levels by flax oil diet with a low n-6/n-3 PUFA ratio. A high-fat diet containing PUFA compared to high SFA diet led to a decreased concentration of TAG in the serum of C57BL/6J mice [51]. However, recently published data show that a high intake of walnut oil did not change plasma TAG which might also be the result of the ApoE deficiency in this model [54].

As opposed to these latter-published data in our study, the drop in hepatic TAG content by high intake of walnut oil was paralleled by a significant elevation of fasting serum TAG in energy-controlled obese rats. In obese ad libitum fed rats, also, the high intake of lard shows this effect. These results might be an indication that besides being substrate for hepatic TAG synthesis, fatty acids are also capable of being signaling molecules directly affecting hepatic receptors (e.g., PPARs), enzymes and transport molecules and also catalyzing hepatocyte VLDL assembly.

It has been established in several studies in human and animal experiments that dietary n-3 PUFA decrease plasma TAG by suppressing hepatic VLDL assembly [60–63]. However, the results presented here let us suggest that a high intake of walnut oil rather increased than decreased VLDL secretion as reflected by elevated plasma concentration of fasting TAG in steatotic animals.

Increasing fasting plasma lipids might be an indication for an elevating insulin resistance characterized by diabetic dyslipidemia [64]. However, in the present study, changes of insulin resistance causing the observed hypertriglyceridemia may be excluded, as plasma insulin and glucose as well as adiponectin were not significantly affected by walnut oil in obese pair fed (data not shown), which showed the strongest modulation of plasma and hepatic lipids by walnut oil. Further, also, the fasting plasma concentration of non-esterified fatty acids (NEFA), liberated from peripheral fat tissue by promoted lipolysis under insulin-resistant circumstances, was not affected by walnut oil intervention (data not shown).

Besides changes in insulin sensitivity, changes in the hepatocyte redox status might be relevant for the liver response to dietary lipid intervention. The pathogenesis of steatosis is mainly characterized by excess accumulation of lipids which also impairs the oxidative capacity of the mitochondria, increasing the reduced state of the electron transport chain complexes and stimulating peroxisomal and microsomal pathways of lipid oxidation [65]. Interestingly, Botham et al. [66] have demonstrated that delivery of n-3 PUFA to hepatocytes objected to minor changes in cellular redox level rather increased than decreased VLDL secretion as observed by n-3 PUFA under normal conditions. Therefore, also, interactions between dietary walnut ingredients and the cellular redox status might have been responsible for the observed unexpected results of an increased hepatic lipid assembly and resulting hyperlipidemia. Similar to our results, attenuation of hepatic steatosis in Zucker obese rats by different dietary constituents have been paralleled by increased fasting plasma TAG [67, 68]. These data are in line with ours and share common features with the basic principle to lower ectopic lipid stores in the liver by increasing hepatic lipid disposal.

As based on this hypothesis of an increased VLDL assembly, we have conducted further attempts to elucidate possible signaling pathways which might be responsible for the observed reduction in hepatic and increase in serum TAG by a high intake of walnut oil in steatotic rodents.

It has been generally accepted that n-3 and n-6 PUFA are key regulators of hepatic gene transcription. However, recent studies have shown that the ability of PUFA regulating the expression of target genes, such as PPAR-α or SREBP1c and ChREBP, together with downstream lipogenic enzymes, e.g., FAS and ACC, might be determined by the kind of fatty acid supplied by a distinct oil [37, 51, 57]. While Yang et al. [51] demonstrated that high intake of DHA and EPA by fish oil reduced the hepatic expression of SREBP1c and the target genes FAS and ACC in C57BL/6J mice [51], a high intake of α-linolenic by flax oil did not affect SREBP1c and PPAR-α in obese rats [2]. These latter observations are in line with ours, since walnut oil with a high amount of α-linolenic acid did not affect the SREBP1c and PPAR-α mRNA expression in obese Zucker rats.

LPL is an enzyme which plays a role in plasma TAG regulation. It promotes plasma TAG clearance by hydrolyzing triglycerides from chylomicrons and VLDL [69, 70]. Previous studies have demonstrated that the development of hypertriglyceridemia is closely associated with the downregulation of LPL activity [71]. In our study in energy-controlled obese pair fed rats, the amount of LPL mRNA was decreased by a high intake compared to a low intake of walnut oil. This result might be independent of the distinct fatty acid pattern of the walnut oil, but mainly determined by the amount of dietary fat irrespective of its dietary source since also the high intake of lard significantly reduced LPL gene expression.

Interestingly, our gene expression data further show that a high load with walnut oil, but not lard significantly increased MTTP gene expression in lean and obese Zucker rats. MTTP exerts a central role in VLDL assembly and secretion [10]. Biosynthesis of hepatic VLDL is critically dependent on the coordinated interactions of apolipoprotein B (apoB) and MTTP [72]. The knock-out of MTTP impairs the assembly and secretion of TAG-rich lipoproteins from the liver [73], while hepatic MTTP overexpression in leptin-deficient mice resulted an increased VLDL secretion and a decreased hepatic TAG content [74]. Summarizing these latter and our current findings, we suggest that the observed MTTP overexpression might in part be responsible for the walnut oil-mediated decrease in hepatic and increase in circulating TAG.

In addition to these results, we also found that the SCD-index is significantly higher in obese compared to respective lean groups. The SCD-index is an indirect indicator for the SCD enzyme activity. This enzyme is the rate-limiting enzyme of hepatic desaturation of stearic acid to oleic acid, and therefore, changes of SCD activity modulate the 18:1/18:0 ratio in hepatocytes. Moreover, elevated hepatic TAG levels and provoked lipogenesis were mainly mediated by upregulation of SCD activity and oleic acid biosynthesis rather than a high supply of dietary oleic acid [75, 76]. Further, hepatic Δ5- and Δ6-desaturases are known to be upregulated by SREBP-1 which was increased in our study in obese as compared to respective lean, too. In addition, we have shown that W14 significantly depressed the SCD-index in steatotic obese, but SREBP1 mRNA levels were not significantly reduced accordingly. Therefore, we suggest that under our experimental conditions of the W14-provoked decrease in hepatic TAG might not be randomly related to, but might be at least in part a consequence of reduced SCD-1 activity and endogenous oleic acid biosynthesis.

Collectively, the results of our study establish that the observed bioactivity of walnut oil can be attributed to multiple metabolic modulations, including an elevated TAG efflux by VLDL assembly. This, in combination with impaired lipolytic conversion of TAG-rich remnants and reduced hepatic oleic acid and subsequent reduced TAG synthesis results in subsequent reduced hepatic and elevated circulating TAG levels.

Besides the walnut oil-mediated modulation of hepatic and circulating TAG concentration, a high walnut-intake reduces total cholesterol as summarized in the framework of a meta-analysis [77], which has been confirmed more recently by a randomized cross-over study in free-living [78] and diabetic subjects [33]. The results derived from feeding studies confirmed the cholesterol-lowering effect of walnuts [79], while rodent data on the cholesterol-lowering effect are more inconsistent, showing either no effect [54] or an increased serum cholesterol concentration after feeding a 5 % (w/w) walnut oil diet in ApoE-deficient hypercholesterolemic mice [80]. Thus, our observations of a cholesterol-lowering effect of high walnut oil are in line with human data. As this effect has not been observed in high lard groups, the cholesterol-lowering effect might be either a result of the PUFA content of walnut oil [81], leading to a decreased cholesterol synthesis or a consequence of walnut oil phytosterols, especially sitosterol and campesterol (Table 3), which share structural similarity with cholesterol and thus reduce serum cholesterol by inhibition of cholesterol absorption [26, 82].

In addition to the reduced serum cholesterol, also, the hepatic cholesterol content is significantly decreased by high walnut oil and lard. Previous studies have shown that PUFA-rich diets suppress SREBP-1 which is a key transcriptional factor involved in up-regulation of cholesterol synthesis [83]. Accordingly, also in our study, the higher hepatic cholesterol content in obese well corresponded to elevated hepatic SREBP-1 mRNA levels in obese compared to the lean groups. However, the SREBP-1 gene expression did not significantly change, and also SREBP-1 target genes FAS and ACC remained constant, while high walnut oil significantly dropped serum cholesterol in all phenotypes. Thus, the observed reduction in serum cholesterol by walnut oil is regulated independently from hepatic SREBP-1 or changes in SREBP-1 expression due to post-transcriptional regulation, e.g., by changing the maturation of SREBP-1 protein, as described previously for PUFA-rich fish oil [83].

Patients with NAFLD have increased circulating level of artherosclerotic biomarkers like sICAM [84–86]. Although classically synthesized and secreted by vascular endothelial cells, Thakur et al. [84] suggested that circulating levels of sICAM might serve as a useful marker of liver injury and steatohepatitis. They observed higher sICAM concentrations in subjects with NAFLD than in lean controls, which is in line with the observations in our animal model. Whether the circulating levels of sICAM also originate from the liver is most likely, as NASH livers express elevated amounts of ICAM mRNA [85]. In addition, Thakur et al. [84] described that the steatotic compared to the normal liver synthesize and probably also secrete elevated amounts of hepatic ICAM-1. Although not being reflected according to significant mRNA changes, our finding that a high intake of walnut oil to a significantly higher extend than lard significantly decreased serum sICAM in parallel with the observed loss of hepatic TAG further promotes the suggestion that circulating sICAM might originate from the liver and also relates to the grade of hepatic steatosis in our model.

In conclusion, our results provide evidence that walnut oil is capable of differentially modifying hepatic and systemic lipid homoeostasis. Moreover, walnut oil also affects endocrine factors associated with artherosclerosis or chronic inflammation. Further, with regard to most parameters relevant to steatosis-associated dyslipidemia, the bioactivity of walnut oil is most probably distinct from that of similar high dose of lard and thus might be the result of the complex mixture of PUFA, but also phytosterols and/or tocopherols might contribute to this bioactivity as also γ-tocopherol has been shown to decrease liver TAG content in a rodent fatty liver model [87]. In this context, it has to be emphasized that whole foods might exert effects beyond those of identified single major ingredients [88] clearly opposing pharmaceutically driven concepts that favor the use of purified fatty acids for drug-based NAFLD intervention, as recently reviewed by Musso et al. [89]. Regarding this general view of favoring complex food instead of individual compounds, also the anti-prostate cancer bioactivity of whole walnut seeds could unexpectedly not be attributed to their fatty acids or tocopherol contents [79]. Perspectively, our work on walnut oil will be extended to the whole walnut seed, too, in order to elucidate the combinatory role of polyphenolic and lipophilic walnut ingredients with respect to their anti-steatotic bioactivity.
